# Elevated [CO_2_] magnifies isoprene emissions under heat and improves thermal resistance in hybrid aspen

**DOI:** 10.1093/jxb/ert318

**Published:** 2013-10-23

**Authors:** Zhihong Sun, Katja Hüve, Vivian Vislap, Ülo Niinemets

**Affiliations:** Institute of Agricultural and Environmental Sciences, Estonian University of Life Sciences, Kreutzwaldi 1, Tartu 51014, Estonia

**Keywords:** BVOCs, foliage traits, future emissions, heat stress, isoprene CO2 response, temperature response.

## Abstract

Isoprene emissions importantly protect plants from heat stress, but the emissions become inhibited by instantaneous increase of [CO_2_], and it is currently unclear how isoprene-emitting plants cope with future more frequent and severe heat episodes under high [CO_2_]. Hybrid aspen (*Populus tremula* x *Populus tremuloides*) saplings grown under ambient [CO_2_] of 380 μmol mol^−1^ and elevated [CO_2_] of 780 μmol mol^−1^ were used to test the hypothesis that acclimation to elevated [CO_2_] reduces the inhibitory effect of high [CO_2_] on emissions. Elevated-[CO_2_]-grown plants had greater isoprene emission capacity and a stronger increase of isoprene emissions with increasing temperature. High temperatures abolished the instantaneous [CO_2_] sensitivity of isoprene emission, possibly due to removing the substrate limitation resulting from curbed cycling of inorganic phosphate. As a result, isoprene emissions were highest in elevated-[CO_2_]-grown plants under high measurement [CO_2_]. Overall, elevated growth [CO_2_] improved heat resistance of photosynthesis, in particular, when assessed under high ambient [CO_2_] and the improved heat resistance was associated with greater cellular sugar and isoprene concentrations. Thus, contrary to expectations, these results suggest that isoprene emissions might increase in the future.

## Introduction

Isoprene is the most abundant reactive volatile hydrocarbon emitted from a wide range of plant species (Fineschi *et al.*, 2013; Monson *et al.*, 2013; Sharkey *et al.*, 2013). As a highly reactive volatile, isoprene significantly influences air quality by participating in ozone-forming reactions, and can also influence climate by participating in secondary organic aerosol formation (Claeys *et al.*, 2004).

Isoprene as a small liphophilic molecule further plays important biological roles in protecting plants from abiotic stresses, in particular conferring greater heat resistance (Possell and Loreto, 2013; Sharkey *et al.*, 2008). Isoprene can directly stabilize biomembranes avoiding excessive fluidity at high temperatures (Sharkey *et al.*, 2001; Singsaas *et al.*, 1997; Siwko *et al.*, 2007), but isoprene can also quench reactive oxygen species formed under heat stress (Affek and Yakir, 2002; Loreto *et al.*, 2001; Vickers *et al.*, 2009*a*, 2009*b*). There are multiple defences against sustained heat stress, including synthesis of polyterpenoids such as zeaxanthin (e.g. Tardy and Havaux, 1997), accumulation of osmotica (e.g. Hüve *et al.*, 2006), and synthesis of heat-shock proteins (e.g. Riezman, 2004). However, rapid synthesis of volatile isoprene is especially advantageous in environments with intermittent heat periods such as those occurring during sunflecks when elicitation of other protective mechanisms is too slow (Behnke *et al.*, 2007, 2013; Niinemets and Monson, 2013; Singsaas *et al.*, 1999; Singsaas and Sharkey, 1998), but leaves may rapidly heat up to temperatures 45–50 °C (Singsaas *et al.*, 1999; Singsaas and Sharkey, 1998; Valladares and Niinemets, 2007).

In plants, isoprene is formed in plastids from its immediate precursor dimethylallyl diphosphate (DMADP) by isoprene synthase (for recent reviews see Li and Sharkey, 2013*b*; Rosenkranz and Schnitzler, 2013; Sharkey *et al.*, 2013). Isoprene emissions increase hyperbolically with increasing light intensity and depend on temperature and ambient CO_2_ concentration according to asymmetric response curves with optima at leaf temperature of ~40–45 °C and at intercellular [CO_2_] of ~100–150 μmol mol^−1^ (Li and Sharkey, 2013*b*; Loreto and Sharkey, 1990; Monson *et al.*, 2012; Sun *et al.*, 2012). Isoprene emission responses to short-term modifications in environmental drivers have been simulated assuming independent controls by different environmental drivers (for recent reviews see Grote *et al.*, 2013; Monson *et al.*, 2012). Based on the instantaneous CO_2_ response curves of isoprene emission, it has been suggested that isoprene emissions will decline in the future due to increases in atmospheric [CO_2_] (e.g. Arneth *et al.*, 2007; Heald *et al.*, 2009; Wilkinson *et al.*, 2009). Such a reduction of isoprene emissions in future atmospheres would imply reduced capacity of plants to cope with recurrent heat episodes by isoprene emission. However, short-term fluctuations in all environmental drivers can modify the size of DMADP pool, and thus the environmental controls on isoprene emission are interactive rather than additive (Li and Sharkey, 2013*a*, 2013*b*; Rasulov *et al.*, 2009*b*, 2010; Sun *et al.*, 2012), suggesting that direct extrapolation based on additive dependencies is not warranted.So far, information on the CO_2_ sensitivity of isoprene emissions under heat stress is limited. Way *et al.* (2011) observed that grey poplar (*Populus* x *canescens*) plants grown and measured at sub-ambient [CO_2_] of 190 μmol mol^−1^ had higher isoprene emission rate than plants grown and measured at elevated [CO_2_] of 590 μmol mol^−1^ at 30–42 °C. On the other hand, Rasulov *et al.* (2010) reported that inhibition of isoprene emission at the measurement [CO_2_] of 800 μmol mol^−1^ relative to 390 μmol mol^−1^ was lost at temperatures higher than 35 °C. Given the strongly non-linear response of isoprene emissions to [CO_2_], these discrepancies might reflect different [CO_2_] contrasts in these studies, and suggest that the sub-ambient compared with elevated [CO_2_] contrast is not appropriate to extrapolate isoprene emission responses to elevated temperatures from current ambient [CO_2_] to future conditions.

Furthermore, isoprene emissions can acclimate to growth [CO_2_] concentration, resulting in altered [CO_2_] sensitivity of isoprene emission as well as in changes in the emission capacity (Calfapietra *et al.*, 2007, 2008, 2013; Sun *et al.*, 2012; Wilkinson *et al.*, 2009). In fact, there is little evidence of downregulation in isoprene emission capacity under elevated [CO_2_], and the emission capacity may even increase in elevated-[CO_2_]-acclimated plants (Li *et al.*, 2009; Sharkey *et al.*, 1991; Sun *et al.*, 2012). Such an elevation of emission capacity may partly compensate for reduction of emissions due to limited DMADP pool size under high ambient [CO_2_], especially under high light (Sun *et al.*, 2012). However, the overall effect of [CO_2_] acclimation on isoprene emissions under high temperatures will depend on temperature-dependent changes in [CO_2_] sensitivity of emissions.

As a further complication, there is evidence of enhanced thermal sensitivity of photosynthetic apparatus in both isoprene- and non-isoprene-emitting species acclimated to elevated [CO_2_] (Darbah *et al.*, 2010; Taub *et al.*, 2000; Way *et al.*, 2011), possibly as the result of enhanced sugar concentrations that stabilize biomembranes (Hüve *et al.*, 2006). There is currently no information on possible modifications of temperature dependencies of isoprene emission by growth [CO_2_] independent of the effects of instantaneous [CO_2_]. This is an important gap needing urgent filling to understand the heat effects on isoprene emissions in plants grown in different [CO_2_] environments as well as to improve modelling of isoprene emissions to future conditions.

In this study, we asked how the acclimation to elevated [CO_2_] alters the heat resistance of photosynthesis and the temperature response of isoprene emission in strong isoprene-emitter hybrid aspen (*Populus tremula* x *Populus tremuloides*). We hypothesized that plants grown under elevated CO_2_ have greater heat tolerance of photosynthetic apparatus and sustain greater isoprene emission rates, especially under supra-optimal temperatures. The results of this study will provide novel insight into the effects of CO_2_ acclimation on isoprene emissions, and into the role of isoprene in thermotolerance in future climates. Although global change is expected to alter moderately average temperatures (Meehl *et al.*, 2007), temperatures strongly fluctuate during the day due to changes in radiation input, occasionally exceeding the threshold for leaf damage during heatflecks (Behnke *et al.*, 2007; Singsaas *et al.*, 1999; Singsaas and Sharkey, 2000; Way *et al.*, 2011). It is these rapid and potentially damaging high-temperature excursions that are expected to become more frequent in climates with overall warmer temperatures. Thus, rapid protection of plant photosynthetic apparatus by adaptive features such as isoprene emission can potentially play a major role in vegetation responses to future climates.

## Material and methods

### Plant material and growth system

For these experiments, 2-year-old saplings of hybrid aspen (*P. tremuloides* Michx. x *P. tremula* L.) clone H200 were used (Rasulov *et al.*, 2009*a*, 2011; Vahala *et al.*, 2003 for details of the genotype). Before the start of the experimental treatments, the saplings were kept in cold room at −2 °C in the dormant state. Dormant plants were planted in 3 L plastic pots filled with sand and peat mixture (1:1), and dormancy was broken by transferring them to a growth room at 20 °C for 4 d. Plants with enlarged buds were installed in the whole-plant open gas-exchange/growth system for different [CO_2_] treatments. During plant growth, supply of nutrients and water was maintained at close to optimal levels (Sun *et al.*, 2012, 2013 for details of plant growth).

The four-chamber whole-plant open gas-exchange/growth system’s design and operation have been described in our earlier studies (Sun *et al.*, 2012, 2013). Briefly, each individual glass chamber had a volume of 12.5 L (diameter 0.2 m, height 0.4 m) to accommodate the entire foliage of a sapling, and the flow rate through the chamber was 7.5 L min^−1^, resulting in a relatively low chamber half-time of 70 s (see Niinemets, 2012 for a comparison of whole-plant gas-exchange systems). Chambers 1 and 3 were kept at the ambient [CO_2_] (mean±SD) of 380±10 μmol mol^−1^, and chambers 2 and 4 were treated with the elevated [CO_2_] of 780±10 μmol mol^−1^. Chamber air temperature was maintained at 28–30/23 °C (day/night) and relative humidity was 60%. Photoperiod length was 12h and the light intensity at the top of the plants was 500 μmol m^−2^ s^−1^ at start of the experiment, increasing to 800 μmol m^−2^ s^−1^ by the end of the experiments when the plants had filled the growth chamber (Sun *et al.*, 2012).

After 30–40 d growth under given conditions when plants had formed a branched canopy filling the growth chamber (Sun *et al.*, 2013 for details) plants were randomly moved out and temperature responses of foliage gas exchange were measured in individual attached fully mature (10–12 d old) leaves. The experiment was replicated four times, altogether with 16 plants in two treatment CO_2_ concentrations.

### Measurements of temperature responses of net assimilation and isoprene emission

A Walz GFS-3000 portable gas-exchange/chlorophyll fluorescence system equipped with a LED array/PAM fluorometer 3055-FL (Walz GmbH, Effeltrich, Germany) and linked with a Fast Isoprene Sensor (Hills-Scientific, Boulder, CO, USA) was used for combined measurements of photosynthetic characteristics and isoprene emission rates as described in detail in Sun *et al.* (2012). The isoprene analyser was calibrated frequently with a standard gas containing 4.47 μmol mol^−1^ isoprene in N_2_ (Hills-Scientific).

The measurements were started by clamping the leaf in the cuvette and establishing the standard conditions of leaf temperature 30 °C, light intensity 500μmol m^−2^ s^−1^, and relative humidity 60%, corresponding to the environmental conditions during plant growth. Temperature responses of net assimilation and isoprene emission were measured after steady-state conditions had been established in the standard conditions at both the growth light intensity of 500μmol m^−2^ s^−1^ and the strong light intensity of 2000μmol m^−2^ s^−1^, and at both CO_2_ concentrations of 380 and 780μmol mol^−1^ using separate leaves for each combination of light and [CO_2_]. We denote the growth [CO_2_] treatments (380 and 780 μmol mol^−1^) as ambient and elevated, and measurement CO_2_ concentrations (380 and 780 μmol mol^−1^) as 380 and 780, yielding four combinations of growth and measurement CO_2_ concentrations: ambient (380), ambient (780), elevated (380), and elevated (780).

During response-curve measurements, leaf temperature was changed from the stabilization temperature of 30 °C to higher temperatures in steps of 5 °C up to 50 °C. The leaf was maintained at every temperature for 8min that was sufficient for establishment of steady-state conditions for measurements under 35–45 °C. This time period is comparable to past studies investigating the effect of heat- flecks on isoprene emission and photosynthesis (Behnke *et al.*, 2007, 2013; Way *et al.*, 2011). Time-dependent reductions in net assimilation rate were observed at 50 °C and sometimes at 45 °C as reported in other studies, likely reflecting time-dependent accumulation of reactive oxygen species and damage to photosynthetic apparatus (Hüve *et al.*, 2006, 2011). Analogous time-dependent decreases can be sometimes observed for isoprene emissions (Loreto *et al.*, 2006; Rasulov *et al.*, 2010; Singsaas and Sharkey, 2000), mostly resulting from time-dependent reductions in the pool size of DMADP, the substrate for isoprene formation (Rasulov *et al.*, 2010). In the case of time-dependent changes, there will be no true steady state and therefore, standardizing the time of sampling is highly recommended (Niinemets *et al.*, 2010*a*, 2010d) as it allows for comparison of all leaves at a common heat dose.

Net assimilation, transpiration, and isoprene emission rates and steady-state fluorescence yield, *F*, were recorded during the last 30 s measurement period at the given temperature. Thereafter, a saturating pulse of white light was given to measure the maximum light-adapted quantum yield of photosystem II (PSII), *F*_m_′. The effective quantum yield of PSII (Φ_PSII_) was determined as (*F*_m_′−*F*)/*F*_m_′.

Isoprene concentration in leaf intercellular air space (*C*_iso,i_) was calculated as:


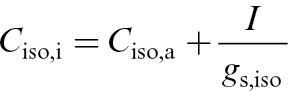
(1)

where *C*_iso,a_ is the isoprene concentration in the leaf chamber, and *g*_s,iso_ is the stomatal conductance for isoprene. *g*_s,iso_ was estimated as the product of stomatal conductance to water vapour and the ratio of the binary diffusion coefficients for isoprene (*D*_iso_) and water vapour (*D*_H2O_) (Niinemets and Reichstein, 2003). The temperature relationships of *D*_iso_ and *D*_H2O_ were developed based on the Chapman and Enskog theory of gas diffusion by intermolecular collision as in Niinemets and Reichstein (2003). The ratio *D*_iso_/*D*_H2O_ was essentially independent of temperature, and an average value of 0.339 was used. The corresponding equilibrium concentration of isoprene in leaf liquid phase (nmol m^−3^) is given as *C*_iso,w_=*C*_iso,i_ *P*/*H*_pc_, where *P* is the air pressure (Pa) and *H*_pc_ is the Henry’s law constant for isoprene. A value of *H*_pc_ of 9950 Pa m^3^ mol^−1^ at 30 °C was estimated from available data at 25 °C assuming an enthalpy of volatilization of 37 kJ mol^−1^ (Copolovici and Niinemets, 2005; Niinemets and Reichstein, 2003).

Overall, photosynthetic capacities (light-saturated net assimilation rate at an ambient CO_2_ concentration of 380 μmol mol^−1^ and leaf temperature of 30 °C) between about 7 to 20 μmol m^−2^ s^−1^, and isoprene emission capacities between about 10 to 40 nmol m^−2^ s^−1^ observed across the leaves measured in plants grown under ambient and elevated CO_2_ concentrations are similar to the values of these traits in *Populus* spp. observed in other studies (Calfapietra *et al.*, 2007; Loreto *et al.*, 2007; Niinemets *et al.*, 2010*c*; Pegoraro *et al.*, 2004; Rasulov *et al.*, 2010, 2011; Rosenstiel *et al.*, 2003; Wiberley *et al.*, 2008, 2009; Wilkinson *et al.*, 2009). Lower values of isoprene emission from poplar species have been reported in some other studies, including emissions from young leaves (Centritto *et al.*, 2004), from shaded leaves (Loreto *et al.*, 2007), and from tissue-cultured plants with extremely thin leaves (Schnitzler *et al.*, 2004). As isoprene synthase content is low in young, shaded, and morphologically weakly developed leaves (Calfapietra *et al.*, 2007; Mayrhofer *et al.*, 2005; Niinemets *et al.*, 2010*c*), lower estimates of isoprene emission rate in these other studies with poplars likely reflect low isoprene synthase activity in these studies. Thus, we conclude that our estimates of isoprene emission capacity are representative of poplar species.

### Estimation of relative changes in net assimilation and isoprene emission rates

To compare the temperature treatment effects independent of differences in the capacities for net assimilation and isoprene emission, we calculated the normalized changes in these traits. Relative change in net assimilation rate, *R*_A_, was calculated as:


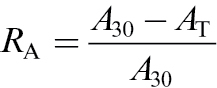
(2)

where *A*_30_ is the net assimilation rate at 30 °C and *A*_T_ that at given temperature *T*. An increase in *R*_A_ reflects a reduction in *A*_T_ compared with *A*_30_. Relative change in the effective quantum yield of PSII was calculated analogously. Relative change in isoprene emission rate due to changes in temperature, *R*_I_, was calculated as:


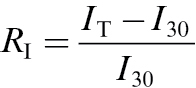
(3)

where *I*_30_ is the isoprene emission rate at 30 °C and *I*_T_ the emission rate at given temperature *T*. An increase in *R*_I_ corresponds to an increase in *I*_T_ relative to *I*_30_. As net assimilation rate generally decreased and isoprene emission rate increased at temperatures above 30 °C, relative changes in net assimilation and isoprene emission were defined differently to have positive values for both *R*_A_ and *R*_I_ across the whole temperature range.

### Electrolyte leakage in response to heat stress

Leaf relative electrolyte leakage, a measure of membrane integrity, was assessed by changes in electrical conductivity of distilled water after soaking the treated leaves (Bajji *et al.*, 2002; Kocheva *et al.*, 2005; Scotti Campos *et al.*, 2003). Detached leaves enclosed in plastic bags were immersed in water at a given temperature (25, 50, and 52 °C) for 5min. Then, three freshly cut discs (7mm in diameter each) from the treated leaf were immediately soaked in 5ml of distilled water at 25 °C. Conductivity of the water was measured in 24h after disc soaking using a conductometer HandyLab LF1 (Schott GmbH, Mainz, Germany). Thereafter, the same flasks with leaf discs were heated in a boiling-water bath for 10min and left to cool for 1h. The solution conductivity was measured again at 25 °C, and the relative electrical conductivity of the sample was expressed as a percentage of the maximum conductivity observed after boiling.

### Foliage morphological and anatomical measurements

After gas-exchange measurements, leaf samples were taken for structural and chemical analyses. Leaf fresh mass and leaf area were determined immediately and dry mass after drying the leaves in a ventilated oven at 70 °C for 48h. Key foliage structural, anatomical, and chemical traits, including leaf dry mass per unit area (*M*_A_), nitrogen and carbon contents, leaf thickness, exposed mesophyll and chloroplast surface area, and number of chloroplasts for leaves developed under the CO_2_ treatments (10–16 replicates per treatment) have been reported by Sun *et al.* (2012, 2013). Elevated-[CO_2_]-grown plants had about 15% thicker leaves with 35% greater *M*_A_ and 20% greater chloroplast exposed surface area per leaf area (Sun *et al.*, 2012). In addition, the cross-sectional area of chloroplasts covered by starch granules per chloroplast area (*a*_chl,s_/*a*_chl_) was about 50% greater under elevated [CO_2_] (Sun *et al.*, 2012). Here we use these data to estimate the distribution of leaf water among different leaf fractions to gain insight into possible differences in leaf sugar distribution and isoprene partitioning among leaf gas and liquid phases. Although water solubility of isoprene is relatively small with the dimensionless Henry’s law constant [*H*_cc_= *H*_pc_/(*RT*_k_), where *R* is the gas constant and *T*_k_ the absolute temperature] at 30 °C being 3.95mol m^−3^ air (mol m^−3^ water), still a significant fraction of whole-leaf isoprene pool can be in the liquid phase depending on the relative size of leaf gas and liquid phases.

First, the volume fraction of mesophyll without intercellular air space (*f*_t,mes_) was calculated as:


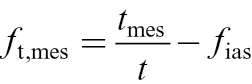
(4)

where *t*_mes_ is the mesophyll thickness, *t* the leaf thickness, and *f*_ias_ is the fraction of intercellular air space. The volume fraction of chloroplasts (*f*_t,chl_) was calculated as the product of *f*_t,mes_ and the ratio of cross-sectional areas of chloroplasts to mesophyll cells (*a*_chl_/*a*_mes_). The fraction of water in leaf mesophyll, *F*_W,mes_, was approximated by:


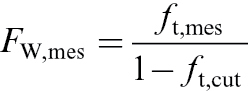
(5)

where *f*_t,cut_ is the volume fraction of cuticle with outer thickened cell walls (Niinemets, 1999). Equation 5 assumes that leaf water is uniformly distributed among epidermis and mesophyll cells. The correction, *f*_t,cut_, was minor for hybrid aspen, but was included for internal consistency. Finally, the volume fraction of water in chloroplasts, *F*_W,chl_, was calculated as:


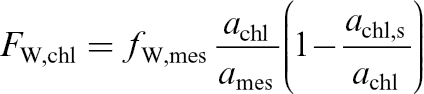
(6)

The second term in this equation, 1−*a*_chl,s_/*a*_chl_, accounts for the reduction of chloroplastic water volume due to presence of starch granules.

### Leaf sugar analysis

Content of soluble sugars was measured with the phenol sulphuric acid method of Dubois *et al.* (1956) as modified by Chow and Landhäusser (2004). The method is based on formation of orange-red colour as the result of condensation of furan derivatives produced under acidic conditions with phenol (Dubois *et al.*, 1956). The soluble sugars were extracted in distilled water at 100 °C for 30min, the extract was treated with the phenol/sulphuric acid reagent as in Chow and Landhäusser (2004) and the absorbance was measured at 485nm with a Shimadzu UV2550PC spectrophotometer (Shimadzu, Kyoto, Japan). The standard curve was developed for sucrose and finally the sugar content was expressed in C_6_ sugar units. Leaf sugar concentration was calculated both per unit leaf dry mass and per unit leaf water.

### Data analyses

[CO_2_] treatment effects on leaf traits were compared by one-way ANOVA followed by Tukey’s test. Within treatments, paired- sample *t*-tests were used to compare the physiological traits measured repeatedly under different measurement light and [CO_2_] conditions. Correlative relationships of *R*_A_ (Eq. 2) versus *R*_I_ (Eq. 3) were analysed by linear regressions and, whenever pertinent, by second-order polynomial regressions. Covariation analyses (ANCOVA) were employed to compare these relationships among the [CO_2_] treatments and at different measurement [CO_2_] and light intensities. In these analyses, the significance of the interaction term (treatment with covariate) was tested first (separate-slope model) and whenever the interaction term was non-significant the model was refitted without the interaction term (common-slope model). SPSS 17.0 (IBM SPSS Statistics) was used for all analyses and all statistical relationships were considered significant at *P*<0.05.

## Results

### Effects of growth [CO_2_] on leaf structure and chemistry

**Fig. 1.**
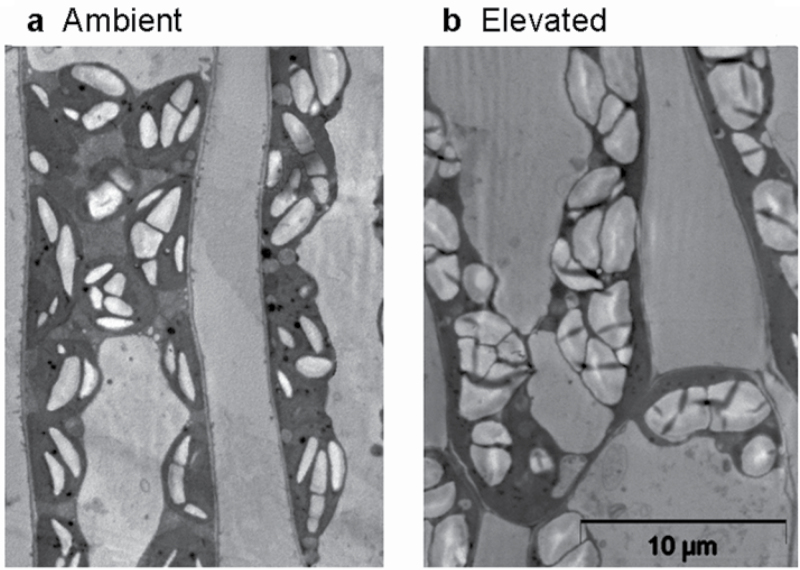
Transmission electron microscopy (TEM) images of leaf palisade mesophyll cells in hybrid aspen (*P. tremula* x *P. tremuloides*) leaves developed under the ambient CO_2_ concentration of 380 μmol mol^−1^ (a) and elevated CO_2_ concentration of 780 μmol mol^−1^ (b). The cells were viewed at 2100× magnification with a Philips Tecnai 10 TEM microscope (FEI, Eindhoven, Netherlands) using an accelerating voltage of 80kV.

As we have demonstrated previously in hybrid aspen (*P. tremula* x *P. tremuloides*), elevated [CO_2_] resulted in thicker leaves with greater leaf dry mass per unit area and more chloroplasts per unit leaf surface area, overall indicating more advanced mesophyll development (Sun *et al.*, 2012, 2013). Here we analyse additional traits with importance in leaf heat resistance (within-leaf distribution of sugars and isoprene). Elevated growth [CO_2_] resulted in greater leaf fresh mass per unit leaf area (*M*_F_) and mass of water per leaf area (*M*_WA_), although there was no significant treatment effect on mass of water per leaf volume (*M*_WV_) (Table 1). The fractions of mesophyll cells and intercellular air space of total leaf volume did not differ among the treatments, but the volume percentage of chloroplasts was higher in elevated-[CO_2_]-grown leaves (Table 1). Although starch granules comprised a greater proportion of chloroplast volume in leaves under elevated [CO_2_] treatment (Fig. 1), the overall fraction of leaf water in chloroplasts (Eq. 6) was higher under elevated [CO_2_] (Table 1). Leaf soluble sugar contents per dry mass (*S*_D_) and per leaf water (*S*_W_) were greater in leaves under elevated [CO_2_] (Table 1).

**Table 1:** *Foliage anatomical, morphological, and chemical traits of hybrid aspen (* *P. tremula x P. tremuloides*) trees grown under ambient (380 μmol mol^−1^) and elevated (780 μmol mol^−1^) atmospheric CO_2_ concentrations

Trait	Treatment		*P*
	Ambient	Elevated	
Leaf fresh mass per unit leaf area (g m^−2^) (*M*_F_)	153.5±3.7	180.9±4.7	<0.0001
Mass of water per leaf area (g m^−2^) (*M*_WA_)	125±5	142.4±3.2	0.001
Mass of water per leaf volume (g cm^−3^) (*M*_WV_)	0.713±0.040	0.728±0.017	0.76
Percentage of intercellular air space (%) (*f*_ias_)	26.2±1.1	24.3±1.1	0.21
Percentage of mesophyll cells of total leaf volume (without air spaces) (%) (*f*_t,mes_, Eq. 4)	59.2±2.1	61.4±3.4	0.25
Percentage of chloroplasts of total leaf volume (without air spaces) (%) (*f*_Chl_)	11.4±1.3	29±5	0.02
Percentage of leaf water in mesophyll (%) (*F*_W,mes_, Eq. 5)	46.1±3.0	51.0±2.4	0.25
Percentage of leaf water in chloroplasts (%) (*F*_W_,_Chl_, Eq. 6)	7.72±0.45	13.0±1.1	0.004
Sugar content in leaf water (g g^−1^) (*S*_W_)	0.036±0.007	0.053±0.008	0.002
Sugar content per dry mass (g g^−1^) (*S*_D_)	0.1293±0.0035	0.1567±0.0033	<0.0001

Data are means±SE of four independent samples (trees). Means were compared using ANOVA. Leaf dry mass per unit area was 28.8±0.6g m^−2^ for plants grown under ambient and 38.6±0.8g m^−2^ for plants grown under elevated [CO_2_] (*P*<0.001) (Sun *et al.*, 2012).

### Dependencies of net assimilation and isoprene emission rates and intercellular isoprene concentration on temperature: general patterns

**Fig. 2.**
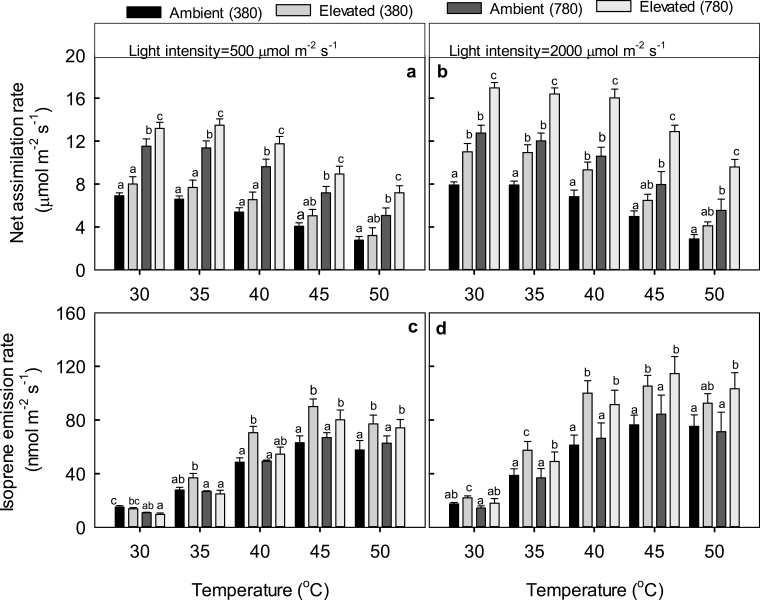
Temperature response of net assimilation rate (a, b), and isoprene emission rate (c, d) in hybrid aspen leaves under different growth and measurement CO_2_ concentrations and at different light intensities. Data in (a) and (c) correspond to measurements under a moderate light intensity of 500 μmol m^−2^ s^−1^ and (b) and (d) to measurements under a strong light intensity of 2000 μmol m^−2^ s^−1^. Ambient (380) and elevated (380) denote plants grown under the ambient [CO_2_] of 380 μmol mol^−1^ and elevated [CO_2_] of 780 μmol mol^−1^, and both measured at [CO_2_] of 380 μmol mol^−1^. Ambient (780) and elevated (780) plants were grown under the ambient [CO_2_] of 380 μmol mol^−1^ and elevated [CO_2_] of 780 μmol mol^−1^, and both measured at [CO_2_] of 780 μmol mol^−1^. Data are means (+SE) of 8–10 replicate leaves. At each individual temperature different letters at the top of each bar indicate statistically significant differences at a given temperature (*P*<0.05).

Net assimilation rate (*A*) of hybrid aspen leaves was the highest at leaf temperatures between 30 and 35 °C (Fig. 2a, b), whereas isoprene emission rate (*I*) increased up to temperatures of 45–50 °C (Fig. 2c, d). Temperature responses were broadly similar under moderately high light intensity of 500 μmol m^−2^ s^−1^ and strong light intensity of 2000 μmol m^−2^ s^−1^ (compare Fig. 2a and b, and Fig. 2c and d).

**Fig. 3.**
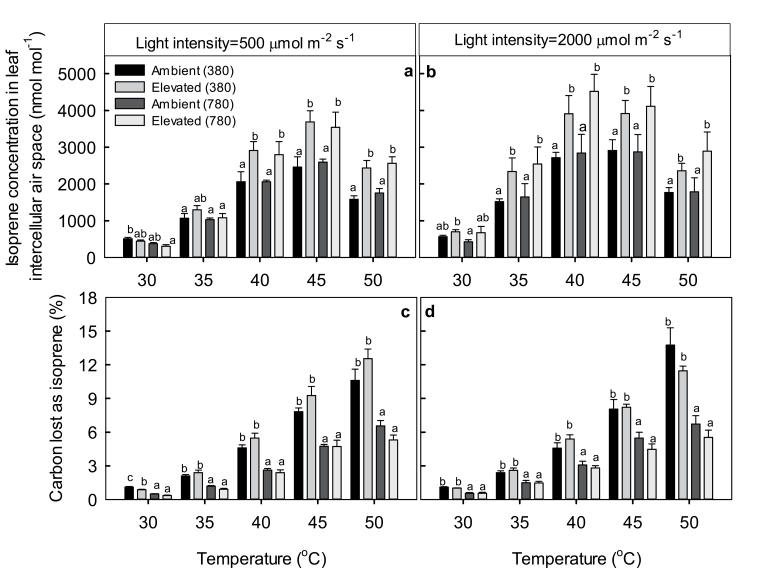
Temperature response of isoprene concentration in leaf intercellular air space (*C*_iso,i_, Eq. 1) (a, b) and the percentage of carbon lost as isoprene (c, d) in hybrid aspen leaves grown under different growth CO_2_ environments and measured under different growth and light conditions. Data are means (+SE) of 8–10 replicate leaves. Data presentation and statistics as in Figure 2.

The temperature dependence of the concentration of isoprene in leaf intercellular air space (*C*_iso,i_) mirrored the temperature response of isoprene emission (Fig. 3a, b), whereas the fraction of carbon lost due to isoprene emission was the highest at 50 °C, reaching up to 15% of net assimilation rate, i.e. almost an order of magnitude increase compared to the carbon lost at 30 °C (Fig. 3c, d).

### Effects of growth [CO_2_] and measurement [CO_2_] and light intensity on net assimilation and isoprene emission rates under different temperatures

Measurement [CO_2_] (instantaneous change in [CO_2_]) generally increased the net assimilation rate (*A*) (Fig. 2a, b), although the increase was weaker for plants grown under ambient [CO_2_] than in plants grown under elevated [CO_2_], especially under moderate light intensity of 500 μmol m^−2^ s^−1^ (see Fig. 2a, b). At the moderate light intensity of 500 μmol m^−2^ s^−1^ and measurement [CO_2_] of 380 μmol mol^−1^, *A* was similar among plants grown at ambient and elevated [CO_2_] (Fig. 2a), but when measured at [CO_2_] of 780 μmol mol^−1^, *A* of elevated-[CO_2_]-grown plants was higher than in ambient-[CO_2_]-grown plants at a given temperature (Fig. 2a). Furthermore, under the strong light intensity of 2000 μmol m^−2^ s^−1^, *A* in elevated-[CO_2_]-grown plants was significantly higher than that in ambient-[CO_2_]-grown plants at both measurement CO_2_ concentrations of 380 and 780 μmol mol^−1^ (Fig. 2b).

Higher measurement [CO_2_] inhibited isoprene emission rate in elevated-[CO_2_]-grown plants at temperatures of 30–35 °C under moderately high light (Fig. 2c) and at 30 °C under strong light (Fig. 2d), but the [CO_2_] inhibition was lost at higher temperatures (Fig. 2c, d). At temperatures higher than 35 °C under moderately high light and higher than 30 °C under strong light, the isoprene emission rate in elevated-[CO_2_]-grown plants exceeded that in ambient-[CO_2_]-grown plants (Fig. 2c, d).

The variations in *C*_iso,i_ among the growth [CO_2_] treatments and measurement [CO_2_] and light intensities reflected the differences in isoprene emission rate (see Fig. 2c, d and Fig. 3a, b). Thus, *C*_iso,i_ was greater at stronger light, did not depend on measurement [CO_2_], and was greater in elevated-[CO_2_]-grown plants above 35 °C under the moderate light intensity of 500 μmol m^−2^ s^−1^ and above 30 °C under the strong light intensity of 2000 μmol m^−2^ s^−1^ (Fig. 3a, b). Given the size of the leaf gas- and liquid-phase pools (Table 1), the predicted amount (concentration multiplied by the volume of a given leaf phase) of isoprene in leaf gas- and liquid-phase pools at 30 °C was roughly similar (0.81-fold lower in the leaf liquid phase for ambient-[CO_2_]-grown plants and 0.89-fold lower for elevated-[CO_2_]-grown leaves). However, given the greater fraction of leaf water in chloroplasts in elevated-[CO_2_]-grown plants (Table 1), the total amount of isoprene associated with chloroplasts was also greater in elevated-[CO_2_]-grown plants.

The fraction of carbon lost as isoprene was always larger at the measurement [CO_2_] of 380 μmol mol^−1^ than at 780 μmol mol^−1^ (Fig. 3c, d). Growth [CO_2_] effects on relative carbon loss were minor, with the only significant difference being the greater carbon loss at 30 °C under moderate light intensity and at measurement [CO_2_] of 380 μmol mol^−1^ in ambient-[CO_2_]-grown plants (Fig. 3c).

### Relationship of assimilation and isoprene emission rate with temperature

**Fig. 4.**
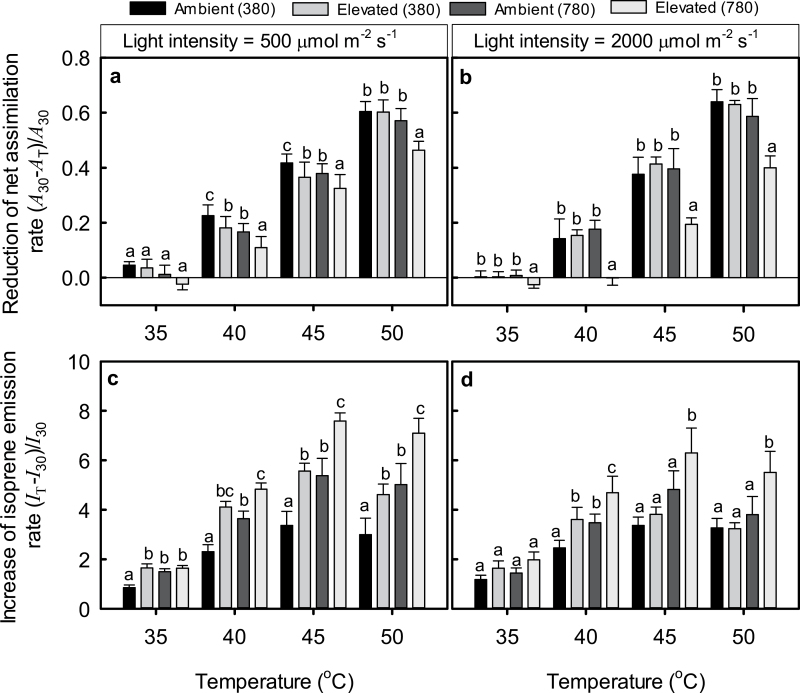
Relative reduction of net assimilation rate (Eq. 2) (a, b) and relative increase of isoprene emission rate (Eq. 3) (c, d) with increasing temperature in hybrid aspen leaves grown under different [CO_2_] of 380 μmol mol^−1^ (ambient) and 780 μmol mol^−1^ (elevated) and measured under different [CO_2_] and light conditions. Measurement CO_2_ concentrations, 380 or 780 μmol mol^−1^, are shown in parentheses for each treatment. Data are means (+SE) of 8–10 replicate leaves.

**Fig. 5.**
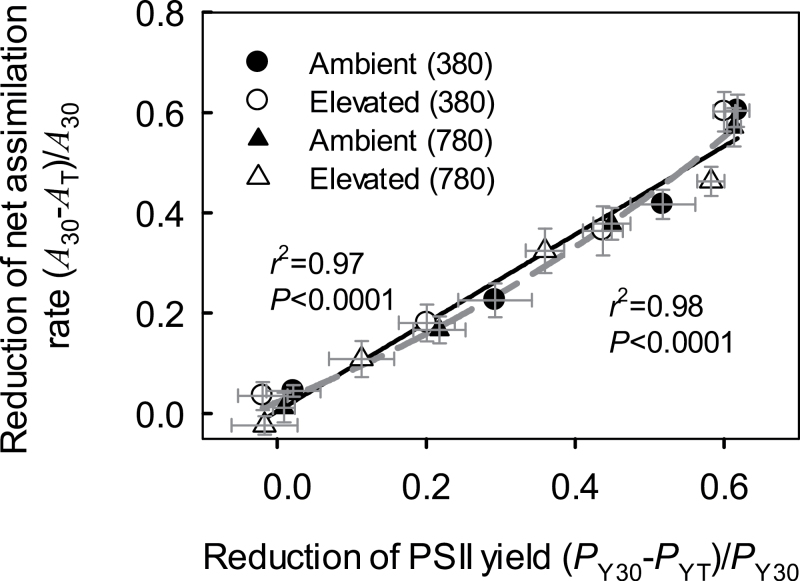
Relationship of the decrease of net assimilation rate (Eq. 2) with the reduction in effective quantum yield of PSII over the temperature range of 30–50 °C (*n*=8–10 for individual data points). Temperature responses of the change in net assimilation rate are illustrated in Figure 4a, c. The measurements conducted at light intensities of 500 and 2000 μmol m^−2^ s^−1^ were pooled. The decrease of PSII quantum yield was calculated as (*Y*_30_−*Y*_T_)/*Y*_30_, where *Y*_30_ is the yield at 30 °C and *Y*_T_ the PSII yield at any other measurement temperature between 30 and 50 °C. Error bars denote ±SE. Data were fitted by linear regression.

The relative reduction in net assimilation rate (Eq. 2, *R*_A_) increased almost linearly over the temperature range of 35–50 °C (Fig. 4a, b; *r* ^2^>0.9 for linear regressions). In contrast, the relative increase of isoprene emission rate (Eq. 3, *R*_I_) tended to be curvilinearly related to temperature, reaching a maximum at ~45 °C (Fig. 4c, d). *R*_A_ at given temperature was lower in elevated-[CO_2_]-grown leaves measured at 780 μmol mol^−1^ (Fig. 4a, b), except at 35 °C under the light intensity of 500 μmol m^−2^ s^−1^ (Fig. 4a). At this light intensity, *R*_A_ of ambient-[CO_2_] grown plants measured at 380 μmol mol^−1^ was greater than that for the rest of the treatments at 40 and 45 °C (Fig. 4a). Reductions in the effective quantum yield of PSII paralleled changes in *R*_A_, being smaller in elevated-[CO_2_]-grown plants measured at [CO_2_] of 780 μmol mol^−1^ than in the other treatments (*P*<0.001). The reductions in the effective PSII quantum yield and in *R*_A_ were strongly correlated across the different measurement conditions and treatments (Fig. 5).

The values of *R*_I_ in ambient-[CO_2_] grown plants measured at [CO_2_] of 380 μmol mol^−1^ and light intensity of 500 μmol m^−2^ s^−1^ were less than for the rest of the treatments (Fig. 4c), while at higher light level this was the case at 40 °C (Fig. 4d). On the other hand, *R*_I_ in elevated-[CO_2_]-grown plants measured at 780 μmol mol^−1^ was consistently higher than for the rest of the treatments (Fig. 4c, d), except for 35 °C at the higher light intensity (Fig. 4d).

**Fig. 6.**
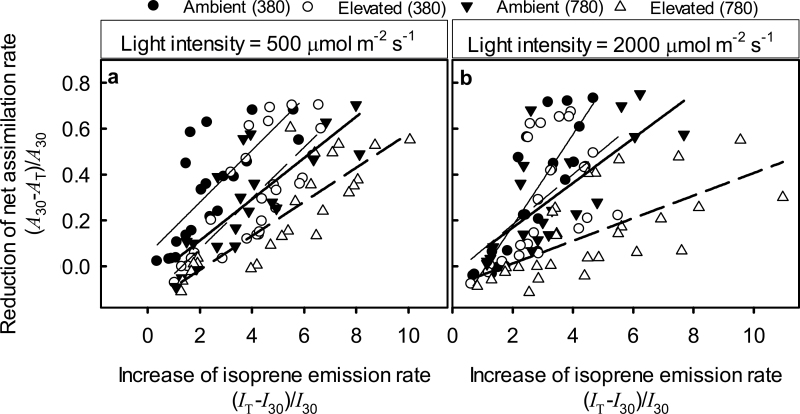
Correlations of the decrease of net assimilation rate (*R*_A_, Eq. 2) with the increase of isoprene emission rate (*R*_I_) during heat stress in hybrid aspen leaves under different growth (ambient of 380 μmol mol^−1^ and elevated of 780 μmol mol^−1^) and measurement [CO_2_] conditions (380 and 780 μmol mol^−1^). The measurements were conducted under a moderate light intensity of 500 μmol m^−2^ s^−1^ (a) and under a strong light intensity of 2000 μmol m^−2^ s^−1^ (b). Linear regression lines are shown to highlight the trends: ambient (380), thin solid line [*r* ^2^=0.56 for (a) and *r* ^2^=0.69 for (b), *P*<0.001 for both]; elevated (380), thin dashed line [*r* ^2^=0.58 for (a) and *r* ^2^=0.28 for (b), *P*<0.0001 for (a) and *P*<0.01 for (b)]; ambient (780), thick solid line [*r* ^2^=0.64 for (a) and *r* ^2^=0.54 for (b), *P*<0.001 for both]; and elevated (780), thick dashed line [*r* ^2^=0.71 for (a) and *r* ^2^=0.43 for (b), *P*<0.001 for both].

The reduction in net assimilation rate was positively correlated with the increase in isoprene emission rate (Fig. 6a, b). For measurements under lower light intensity of 500 μmol m^−2^ s^−1^, the interaction of *R*_I_ with treatment was not significant (*P*>0.8). According to the common-slope ANCOVA, ambient-[CO_2_]-grown leaves measured at 380 μmol mol^−1^ had greater *R*_A_ at given *R*_I_ than that in the other treatments, while elevated-CO_2_-grown leaves measured at 780 μmol mol^−1^ had lower *R*_A_ at given *R*_I_ than that in the other treatments (Fig. 6a; *P*<0.001 for both comparisons). At higher light of 2000 μmol m^−2^ s^−1^ the interaction term was significant, indicating that the slope for elevated-[CO_2_]-grown plants measured at 780 μmol mol^−1^ was less than that for the other growth and measurement [CO_2_] combinations (Fig. 6b; *P*<0.001).

### Membrane leakiness in relation to growth [CO_2_] and temperature-dependent reduction in net assimilation rate

**Fig. 7.**
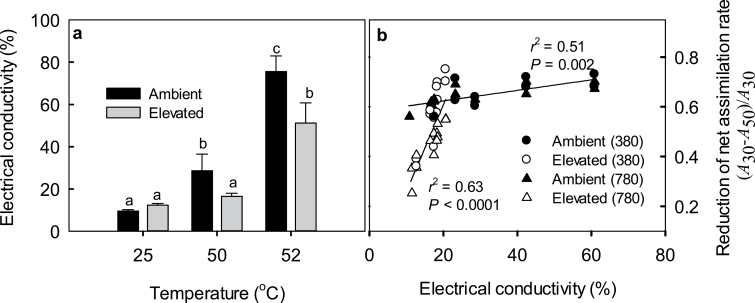
Mean (+SE) relative leaf electrolyte leakage in response to heat stress (a) and correlations of the decrease of net assimilation rate (see Fig. 4a, c) with leaf electrical conductivity at 50 °C (b) in hybrid aspen leaves grown under ambient [CO_2_] of 380 μmol mol^−1^ and elevated [CO_2_] of 780 μmol mol^−1^. In (a) the data are means of 8–10 replicate leaves and means with different letters are significantly different at *P*<0.05 according to one-way ANOVA. In (b) the data correspond to individual measurements, data labels are as in Fig. 5, and the measurements conducted at light intensities of 500 and 2000 μmol m^−2^ s^−1^ were pooled. Data in (b) were fitted by linear regressions.

Relative electrical conductivity, the measure of membrane leakiness, was not significantly increased in elevated-[CO_2_]-grown plants after exposure of leaf discs to 50 °C (Fig. 7a). In contrast, in ambient-[CO_2_]-grown plants, exposure to 50 °C resulted in a significant increase in membrane leakiness (Fig. 7a). Exposure to severe heat stress of 52 °C resulted in enhanced membrane leakiness for both [CO_2_] treatments, but the leakiness was greater for ambient-[CO_2_]-grown plants (Fig. 7a).

Relative electrical conductivity at 50 °C was correlated with the reduction in net assimilation rate (Fig. 7b) and PSII quantum yield (data not shown). However, the slope of this relationship was shallower in ambient-[CO_2_]-grown plants (*P*<0.001 for the interaction term of electrical conductivity × growth [CO_2_]; Fig. 7b), indicating that in ambient-[CO_2_]-grown plants a given reduction in net assimilation rate observed immediately at the end of the exposure period was associated with greater electrolyte leakage over the following 24h soaking of leaf discs.

## Discussion

### Elevated-[CO_2_]-driven modifications in leaf chemistry, structure, and photosynthesis

Elevated growth [CO_2_] resulted in greater leaf fresh mass per unit leaf area (*M*_F_), mass of water per leaf area (*M*_WA_), and fraction of water in chloroplasts (*F*_W,Chl_) (Table 1). However, the mass of leaf water per leaf volume was not significantly different among the treatments, indicating that greater mass of water per leaf area resulted from thicker leaf mesophyll in elevated-[CO_2_]-grown plants (Sun *et al.*, 2012), as has been consistently observed (e.g. Miyazawa *et al.*, 2011; Sims *et al.*, 1998*a*, 1998*b*), and suggested to reflect morphological ‘upregulation’ (Luo *et al.*, 1997).

Elevated [CO_2_] also resulted in greater starch grain number and size inside the chloroplasts (Fig. 1) and enhanced leaf sugar content per dry mass and concentration in leaf water (Table 1), as has been demonstrated in numerous studies (see Makino and Mae, 1999 for reviews; Saxe *et al.*, 1998). Furthermore, greater fraction of leaf water in chloroplasts in elevated-[CO_2_]-grown plants (Table 1) further suggests that a greater fraction of leaf sugar and liquid-phase isoprene is associated with chloroplasts in elevated-[CO_2_]-grown than in ambient-[CO_2_]-grown plants.

Elevated [CO_2_] is often associated with ‘downregulation of photosynthesis’, defined as reduced photosynthesis observed at the same given ambient [CO_2_] (Curtis and Wang, 1998; Johnson, 2006; Luo *et al.*, 1997; Nowak *et al.*, 2004). This downregulation is mainly associated with reduced nitrogen content and may also reflect feedback-inhibition of photosynthesis due to enhanced sugar concentrations (Curtis and Wang, 1998; Jeannette *et al.*, 2000; Johnson, 2006; Luo *et al.*, 1997; Myers *et al.*, 1999; Nowak *et al.*, 2004). However, in our study at optimum nutrient supply we actually observed enhanced photosynthetic capacity in elevated-[CO_2_]-grown plants (Sun *et al.*, 2012; Fig. 2b), indicating no downregulation nor stronger feedback inhibition despite higher sugar concentrations.

### Temperature responses of net assimilation and isoprene emission under different environmental conditions

Isoprene is formed in chloroplasts by isoprene synthase from its immediate precursor dimethylallyl diphosphate (DMADP) (see Li and Sharkey, 2013*b* for a recent review). The major source of chloroplastic DMADP is the plastidic 2-*C*-methyl-d-erythritol 4-phosphate (MEP) pathway that starts with condensation of pyruvate and glyceraldehyde 3-phosphate (GAP) (Lichtenthaler, 1999; Schwender *et al.*, 1997). Although there may be some contribution of the cytosolic mevalonic acid pathway because isopentenyl diphosphate, the isomer of DMADP, may be transferred between the cytosol and plastid, mevalonic acid pathway contribution is generally minor (Bick and Lange, 2003; Laule *et al.*, 2003).

As both the isoprene synthase and the main pathway for DMADP formation are in chloroplasts, and chloroplastic DMADP formation under non-stressed conditions mainly relies on recently fixed carbon—in particular, on primary photosynthetic metabolite GAP—isoprene emission is strongly associated with photosynthetic carbon metabolism (for recent reviews see Li and Sharkey, 2013*b*; Monson, 2013). Pyruvate for DMADP synthesis is assumed to be of cytosolic origin and transported to the chloroplasts in the form of phosphoenolpyruvate (PEP) by a PEP transporter in exchange for inorganic phosphate (P_i_) (Li and Sharkey, 2013*b*; Monson, 2013), but there is also evidence that pyruvate can be formed in chloroplasts from 2-phosphoglycerate (see Rasulov *et al.*, 2011 for a discussion). At any rate, ^13^C-labelling experiments demonstrate that in unstressed plants 85–90% of the carbon in isoprene is derived from recently assimilated photosynthates (Delwiche and Sharkey, 1993; Funk *et al.*, 2004; Karl *et al.*, 2002). However, there are important discrepancies among isoprene emission and photosynthesis as demonstrated by our study and past observations, indicating significant differences in the regulation of isoprene emission and net assimilation rates: (i) the optimum temperature for isoprene emission is greater than that for net assimilation (Fig. 2; see also e.g. Harley *et al.*, 1996; Niinemets *et al.*, 1999) and, as the result, the fraction of carbon lost due to isoprene emission increases at higher temperatures (Fig. 3c, d); (ii) isoprene emission more strongly responds to light than net assimilation rate and is saturated at greater light intensity (Fig. 2 and 3; see also e.g. Harley *et al.*, 1996; Monson *et al.*, 2012; Niinemets *et al.*, 1999, 2010*d*); and (iii) isoprene emission rate is inhibited by above-ambient [CO_2_] concentrations, while net assimilation rate increases (Fig. 2; see Li and Sharkey, 2013*b*; Monson, 2013 for reviews). In fact, isoprene emission is even suppressed at current ambient [CO_2_] relative to sub-ambient [CO_2_] (e.g. Guidolotti *et al.*, 2011; Niinemets *et al.*, 2010*b*; Wilkinson *et al.*, 2009). On the other hand, acclimation of capacities for photosynthesis and isoprene emission to growth [CO_2_] seems to occur in similar manner. When there is a downregulation in photosynthetic capacity in elevated-[CO_2_]-grown leaves, isoprene emission capacity is often reduced as well; in contrast, when photosynthetic capacity is enhanced upon acclimation to elevated [CO_2_], isoprene emission capacity seems to increase as well (see Sun *et al.*, 2012 for a literature review of case studies). In Sun *et al.* (2012) this enhanced emission capacity became evident by increased isoprene emission rate at 30 °C under high light intensity of 2000 μmol m^−2^ s^−1^ and our study further demonstrates that this enhancement is maintained over the entire temperature response (Fig. 2d).

Apart from these general observations, our study demonstrates several important novel aspects of environmental responses of isoprene emission under high temperature and in plants developed in different atmospheric [CO_2_]: (i) the CO_2_ sensitivity of isoprene emission was lost at temperatures higher than 35–40 °C (Fig. 2c, d); (ii) as a result of the loss of [CO_2_] sensitivity of emissions, isoprene emission rates in elevated-[CO_2_]-grown plants exceeded the emissions in ambient-[CO_2_]-grown plants at higher temperatures of 40–50 °C; and (iii) high-temperature emission enhancement was maintained not only at high but also at moderate light intensity (Fig. 2c, d, Fig. 4c, d). In the following, we analyse the possible factors responsible for the loss of CO_2_ sensitivity at higher temperatures.

### Loss of CO_2_ sensitivity of isoprene emission at elevated temperatures

Given that the reduction of isoprene emission at higher measurement [CO_2_] is associated with reduction in chloroplastic DMADP pool size (Li and Sharkey, 2013*b*; Rasulov *et al.*, 2009*b*; Wilkinson *et al.*, 2009), loss of CO_2_ sensitivity at higher temperatures suggests that DMADP should have become more readily available under high [CO_2_] and temperature. Three hypotheses have been offered to explain why DMADP pool size is reduced under high [CO_2_]. According to the first hypothesis, transport of PEP to chloroplasts by PEP/P_i_ antiporter becomes limited at higher [CO_2_] due to a reduction of cytosolic PEP level by faster reaction of PEP carboxylase; this reduces pyruvate availability for DMADP synthesis and ultimately chloroplastic DMADP pool size (Rosenstiel *et al.*, 2003; Wilkinson *et al.*, 2009). However, it is difficult to explain the lack of [CO_2_] sensitivity at higher temperature by this mechanism as PEP carboxylase activity is expected to increase at higher temperature, thereby suppressing cytosolic PEP concentration even more.

Alternatively, Li and Sharkey (2013*b*) suggested that reduction of chloroplastic P_i_ due to feedback limitation of photosynthesis under high [CO_2_], i.e. inability of starch and sucrose synthesis reactions to keep up with synthesis of triose phosphates, especially at lower temperature, reduces PEP/P_i_ transport activity and thereby leads to reduced chloroplastic PEP levels. The third hypothesis was based on the observations that feedback inhibition of photosynthesis is also associated with a reduction of ATP synthesis rate (Sharkey, 1985; Socias *et al.*, 1993). Thus, decreases in ATP availability have been suggested to be responsible for the reduced rate of DMADP formation (Rasulov *et al.*, 2009*b*). As cytosolic sucrose synthesis very strongly responds to temperature (Sage and Sharkey, 1987), increased sucrose synthesis reduces triose phosphate concentrations and increases equilibrium P_i_ concentrations, thereby enhancing both PEP transporter activity (Li and Sharkey, 2013*b*), but also ATP synthesis rate.

However, Way *et al.* (2011) did not observe higher-temperature reduction of inhibition of isoprene emission by [CO_2_] of 590 μmol mol^−1^ compared with sub-ambient level of 190 μmol mol^−1^. The discrepancy among our results and the study of Way *et al.* (2011) likely reflects the strong non-linearity in the [CO_2_] responses of DMADP pool size and isoprene emission. Due to this non-linearity, in relative terms, temperature enhancement of sucrose synthesis rate cannot release as much P_i_ for sub-ambient versus supra-ambient (Way *et al.*, 2011) than for ambient versus supra-ambient measurement [CO_2_] contrast.

On the other hand, emissions at a higher temperature consume much larger amounts of carbon, both in absolute terms and relative to net assimilation rate, than emissions at lower temperatures (Fig. 3c, d). This increase, of almost an order of magnitude, is large enough that it could significantly reduce triose phosphate concentrations and thereby partly restore chloroplastic P_i_ level. However, the situation may be further complicated by the onset of the use of alternative carbon sources. It has been reported that the percentage of carbon derived from recently assimilated photosynthates is reduced under heat stress (Funk *et al.*, 2004). Analogously, drought-stress experiments indicate that even complete inhibition of photosynthesis by severe stress moderately inhibits isoprene emission as isoprene supply can be maintained on the basis of alternative ‘older’ carbon sources not readily labelled by ^13^C (Brilli *et al.*, 2007; Karl *et al.*, 2002; Li and Sharkey, 2013*b*; Schnitzler *et al.*, 2004; Wolfertz *et al.*, 2003). As already suggested in the other studies under drought stress, we hypothesize that such an ‘older’ carbon source for enhanced isoprene emissions under heat stress is most likely chloroplastic starch. Use of old or temporary stored photosynthates through the pentose phosphate pathway or glycolysis could supply the substrates of GAP and pyruvate for DMADP formation. The pentose phosphate pathway is an alternative route for the use of stored photosynthates, generating NADPH and sugar phosphates, particularly in stressed plants (Eicks *et al.*, 2002; Fettke *et al.*, 2011). In fact, heat stress triggers starch hydrolysis (e.g. Hüve *et al.*, 2012), and thus could compensate for possible heavier competition for triose phosphates by enhanced rate of sucrose synthesis. In this study, elevated-[CO_2_]-grown plants had higher soluble sugar and starch contents (Table 1, Fig. 1), and thus more readily available alternative carbon sources can partly explain their higher isoprene emission rates at high temperatures.

### Heat-stress resistance under different growth [CO_2_] and the role of isoprene

High temperature results in excessive membrane fluidity and membrane leakiness, loss of compartmentalization, and reduction of physiological functions such as photosynthetic decline (Balogh *et al.*, 2011; Hald *et al.*, 2008) as was also observed in our study at 50 °C in ambient-[CO_2_]-grown and at 52 °C in elevated-[CO_2_]-grown plants (Fig. 7a). Greater heat resistance in elevated-[CO_2_]-grown plants is in agreement with several past studies (Darbah *et al.*, 2010; Taub *et al.*, 2000; Way *et al.*, 2011). This enhancement is common in both non-isoprene- and isoprene-emitting species, but isoprene-emitting species seem to have higher heat-stress resistance (Darbah *et al.*, 2010; Way *et al.*, 2011). Electrolyte leakage, an integrated estimate over 24h following heat stress, was correlated with the reduction in net assimilation rate, but the slope differed among elevated- and ambient-[CO_2_]-grown plants. We suggest that this is indicative of greater damage at the given short-term reduction in net assimilation rate. In fact, once the stress threshold has been reached, there is a time-dependent reduction in net assimilation rate even after return to lower temperature (Hüve *et al.*, 2011).

The enhancement of thermal tolerance under elevated growth [CO_2_] has been associated with greater sugar concentrations that stabilize membranes under stress (Livingston *et al.*, 2009; Nagao *et al.*, 2005). In our study, elevated growth [CO_2_] resulted in greater leaf sugar content in leaf water (Table 1). However, the overall effect of sugars on heat tolerance may depend on the subcellular distribution of sugars. Given the distribution of water within the leaf, greater proportion of sugars was associated with chloroplasts under elevated [CO_2_], and thus, sugar concentrations were likely elevated both in the cytosol and in the chloroplasts in elevated-[CO_2_]-grown plants. Thus, higher sugar concentrations seemed to contribute to the higher thermal tolerance by maintaining lower membrane leakage of hybrid aspen plants grown under elevated [CO_2_].

In addition to sugars, isoprene, a liphophilic and highly reactive molecule, participates in protecting plants under heat stress. Isoprene has been hypothesized to (i) stabilize and protect membranes against high temperature (Sharkey *et al.*, 2001; Singsaas *et al.*, 1997; Velikova *et al.*, 2011), (ii) serve as antioxidant, eliminating reactive oxygen species produced by heat stress (Possell and Loreto, 2013), and (iii) consume excess energy, especially under high light (Sanadze, 2004, 2010). Given the enhanced isoprene emissions (Fig. 2c, d) and greater intercellular isoprene concentrations in elevated-[CO_2_]-grown plants (Fig. 3a, b) that were also associated with a greater liquid-phase pool of isoprene (see Table 1 for the distribution of leaf water), higher heat-stress resistance of these plants can at least partly be attributed to enhanced isoprene production. Clearly, the stronger the temperature-dependent reduction in net assimilation rate the greater the enhancement of isoprene emission rate (Fig. 6). This uncoupling of isoprene emissions from photosynthesis at temperatures high enough to lead to severe reductions in net assimilation rates is consistent with the involvement of isoprene in heat protection.

Nevertheless, there was a certain mismatch between the reductions in net assimilation rate and increases in isoprene emission rate at 45–50 °C (Fig. 4c, d), suggesting that isoprene emissions themselves became limited by excess temperatures. Such a limitation was less evident for elevated-[CO_2_]-grown plants at higher measurement [CO_2_] of 780 μmol mol^−1^ (Fig. 4c, d). However, different levels of protection could be achieved by given enhancement of isoprene emission rate (Fig. 6), questioning the direct involvement of isoprene in heat protection. Nevertheless, photosynthesis clearly was more stable in elevated-[CO_2_]-grown plants measured at their corresponding growth [CO_2_], and this was accompanied by higher intercellular isoprene concentration (Fig. 3a, b). Thus we suggest that both isoprene and sugars are involved in heat-stress resistance and that differences in the stability of photosynthesis under heat stress at given isoprene emission rate possibly reflect differences in the basal level of heat tolerance provided by sugars.

## Conclusions

The results of this study demonstrate that heat resistance of hybrid aspen was strongly enhanced by elevated growth [CO_2_] and this was associated both with more stable net assimilation rates and particularly strong enhancement of isoprene emissions under heat stress. The evidence of loss of CO_2_ inhibition of isoprene emission at higher temperatures as well as maintenance of enhanced isoprene emission capacity in elevated-[CO_2_]-grown plants, both under moderately elevated temperatures that may be experienced during heat waves and under temperatures resulting in severe heat stress that can occur upon exposure to heatflecks during the day (Fig. 2c, d; Li *et al.*, 2011; Li and Sharkey, 2013*b*; Rasulov *et al.*, 2010), potentially has major implications for predicting isoprene emissions in future climates. Contrary to past suggestions, our results suggest that isoprene might protect leaf photosynthetic function against heat stress more effectively under future elevated [CO_2_] conditions.

## Abbreviations:

DMADP: dimethylallyl diphosphate
GAP: glyceraldehyde 3-phosphate
PEP: phosphoenolpyruvate
P_i_: inorganic phosphate
PSII: photosystem II.
